# Host-specific *Dactylogyrus* parasites revealing new insights on the historical biogeography of Northwest African and Iberian cyprinid fish

**DOI:** 10.1186/s13071-017-2521-x

**Published:** 2017-11-28

**Authors:** Andrea Šimková, Michal Benovics, Imane Rahmouni, Jasna Vukić

**Affiliations:** 1 Department of Botany and Zoology, Faculty of Science, Masaryk University, Kotlářská 2, 611 37 Brno, Czech Republic; 2 Laboratory of Biodiversity, Ecology and Genome, Faculty of Sciences, Mohammed V University in Rabat, Ibn Batouta 4, 1014 RP Rabat, Morocco; 3 Department of Ecology, Faculty of Science, Charles University, Viničná 7, 128 44 Prague 2, Czech Republic

**Keywords:** Cyprinids, *Dactylogyrus*, Historical biogeography, Host specificity, Iberia, Northwest Africa

## Abstract

**Background:**

Host specificity in parasites represents the extent to which a parasite’s distribution is limited to certain host species. Considering host-specific parasites of primarily freshwater fish (such as gill monogeneans), their biogeographical distribution is essentially influenced by both evolutionary and ecological processes. Due to the limited capacity for historical dispersion in freshwater fish, their specific coevolving parasites may, through historical host-parasite associations, at least partially reveal the historical biogeographical routes (or historical contacts) of host species. We used *Dactylogyrus* spp., parasites specific to cyprinid fish, to infer potential historical contacts between Northwest African and European and Asian cyprinid faunas. Using phylogenetic reconstruction, we investigated the origin(s) of host-specific *Dactylogyrus* spp. parasitizing Northwest African and Iberian cyprinid species.

**Results:**

In accordance with hypotheses on the historical biogeography of two cyprinid lineages in Northwest Africa, Barbini (*Luciobarbus*) and Torini (*Carasobarbus*), we demonstrated the multiple origins of Northwest African *Dactylogyrus*. *Dactylogyrus* spp. of *Carasobarbus* spp. originated from Asian cyprinids, while *Dactylogyrus* spp. of *Luciobarbus* spp. originated from European cyprinids. This indicates the historical Northern route of *Dactylogyrus* spp. dispersion to Northwest African *Luciobarbus* species rather than the Southern route, which is currently widely accepted for *Luciobarbus*. In addition, both Northwest African cyprinid lineages were also colonized by *Dactylogyrus marocanus* closely related to *Dactylogyrus* spp. parasitizing African *Labeo* spp., which suggests a single host switch from African Labeonini to Northwest African *Luciobarbus*. We also demonstrated the multiple origins of *Dactylogyrus* spp. parasitizing Iberian *Luciobarbus* species. One Iberian *Dactylogyrus* group was phylogenetically closely related to *Dactylogyrus* of Moroccan *Carasobarbus*, while the second was related to *Dactylogyrus* of Moroccan *Luciobarbus*.

**Conclusions:**

Our study confirms the different origins of two Northwest African cyprinid lineages. It suggests several independent historical contacts between European Iberian *Luciobarbus* and two lineages of Northwest African cyprinids, these contacts associated with host switches of *Dactylogyrus* parasites.

## Background

Primary freshwater fish are supposed to be intolerant to salinity, and thus their dispersal is restricted to freshwater routes only. Because of such limited dispersion mechanisms, relationships between fish lineages may reflect relationships between different areas; therefore, freshwater fish are suitable for studies of historical biogeography [1]. Over evolutionary time, the diversity of parasite communities of such freshwater fish is shaped by coevolutionary and historical biogeographical processes (e.g. [2, 3]). However, over ecological time, parasite biogeography is also influenced by the temporal and spatial variability in ecological factors [4, 5].

Concerning freshwater fish, the biogeography of their helminth parasites was shown to reflect historical processes related to the current distribution of their hosts. For example, helminth diversity in Mexican freshwater fishes is determined by the historical and contemporary biogeography of their hosts [6]. The distribution of the metazoan parasites of the sturgeon fish (Acipenseridae) was shown to be in accord with the historical biogeographical routes of these fishes [7].

The host specificity of fish parasites (i.e. the extent to which a parasite’s distribution is limited to certain host species) seems to be their most important characteristic, with the potential to reflect historical host-parasite associations and to indicate the historical biogeographical routes of hosts. McDowell [8] showed that parasites not coevolving with their galaxioid fish hosts (i.e. Galaxiidae and Retropinnidae) do not support a vicariance biogeography for galaxioid fish. However, if the host specificity of a parasite group is high, then the phylogenetic and biogeographical relationships between hosts and parasites may be mutually illuminating [1].

Gill monogeneans of the highly diversified genus *Dactylogyrus* Diesing, 1850 are species-specific to their cyprinid host species (with some rare exceptions). According to Šimková et al. [9], *Dactylogyrus* species often exhibit strict host specificity (i.e. they are specific to a single cyprinid species), congeneric host specificity (i.e. they are specific to congeneric cyprinid species), or phylogenetic host specificity (i.e. they are specific to phylogenetically closely related cyprinid species). The distribution of *Dactylogyrus* species on their cyprinid hosts reflects the evolutionary history of these fishes [10]. The evolution of *Dactylogyrus* lineages is associated with different cyprinid lineages, and the presence of the same *Dactylogyrus* species on the representatives of different cyprinid lineages (i.e. in cyprinid species with high divergence but living in the same biogeographical area) is only accidental [9, 10]. Little is known about *Dactylogyrus* of cyprinid species living in the Mediterranean region. However, some studies are suggesting that due to high host specificity, the endemism of *Dactylogyrus* parasites follows the endemism of their cyprinid host species. Such endemic *Dactylogyrus* were documented for *Luciobarbus* Heckel, 1843 from the Iberian Peninsula [11], for *Luciobarbus* from Northwest Africa [12], and for cyprinids living in Lake Mikri Prespa (northern Greece) [13, 14].

Cyprinids are primarily freshwater fish with their native distribution in Europe, Asia, Africa and North America. The different cyprinid lineages exhibit different biogeographical distributions across continents [15]. One of the lineages, the subfamily Cyprininae, was recently revised by Yang et al. [16] to include 11 tribes. Most representatives of this subfamily inhabit waters of southern Eurasia and Africa. Of the four evolutionary lineages (i.e. tribes) of Cyprininae present in Africa, two have been recognized in Northwestern Africa. The first lineage includes hexaploid genera of large-sized barbels (*Carasobarbus* Karaman, 1971, *Pterocapoeta* Günther, 1902 and *Labeobarbus* Rüppel, 1835) belonging to the tribe Torini (this tribe includes large-sized barbels from Asia and Africa). The second lineage is represented by tetraploid *Luciobarbus* belonging to the tribe Barbini (this tribe includes the taxa distributed in Eurasia and Northwest Africa). Different origins and different dispersal events from Eurasia to Africa were proposed for these lineages. The two genera which are widespread in Northwest Africa, *Carasobarbus* and *Luciobarbus*, have disjunct distributions. *Carasobarbus* is distributed in Northwest Africa and the Middle East, while *Luciobarbus* is distributed in West Asia, Northwest Africa, Greece and the Iberian Peninsula. The large-sized African hexaploids are not monophyletic like the Moroccan *Carasobarbus* cluster with Middle East *Carasobarbus*, suggesting that the diversification of African hexaploids preceded the separation between the Middle East and Northwest African hexaploids [16, 17]. Tsigenopoulos et al. [17] suggested that the large hexaploids invaded Africa through the land bridge between Africa and Asia (via the Arabian tectonic Plate) formed in the Middle Miocene (about 13 MYA). Using molecular calibration, they calculated that the splitting of the African hexaploids from their Asian ancestors and subsequently the beginning of the diversification of the African hexaploid lineage occurred in the Late Miocene. The genus *Luciobarbus* is paraphyletic, as the clade also includes the genus *Capoeta* Güldenstädt, 1773. Concerning *Luciobarbus* species in Northwest Africa, they do not form a monophyletic group either, as two Northwest African species cluster with Iberian species [16, 18–20]. Concerning *Luciobarbus* in the Iberian Peninsula, three main hypotheses were proposed for their origin; some of them have direct implications for the origin of this genus in Northwest Africa. First, Banarescu [21] and Almaça [22] proposed that the Iberian Peninsula was colonized from the North before the formation of the Pyrenees. Based on this hypothesis, barbels from the Iberian Peninsula are evolutionarily closer to European and African barbels than to Asian species. Secondly, Doadrio [23] proposed that *Luciobarbus* colonized Iberia from Africa via southern Spain at the Miocene-Pliocene boundary (about 5 MYA) after the Messinian salinity crisis of the Mediterranean Sea. Following this hypothesis, barbels from the Iberian Peninsula are phylogenetically closer to Asian and North African barbels than to those of central Europe. Thirdly, Bianco [24] proposed that the distribution of *Luciobarbus* be explained by the freshwater phase (the so-called Lago Mare phase) of the Mediterranean Sea, which supposedly followed the Messinian salinity crisis. Following this hypothesis, Iberian barbels are more related to those of the Balkans than to central European species. However, this third hypothesis has been rejected by many authors by both geological data and the estimation of the time of diversification of freshwater fish species [25] according to the finding of fossils preceding the given geological period. Tsigenopoulos et al. [26] and Yang et al. [16] showed that most *Luciobarbus* species from Northwest Africa are more closely related to *Luciobarbus* from the Middle East than to *Luciobarbus* from the Iberian Peninsula.

The aim of this study was to reconstruct the phylogeny of gill parasites of the genus *Dactylogyrus*, monogeneans specific to cyprinid fish species, to (i) investigate the phylogenetic position of African *Dactylogyrus* parasites in relation to European and Asian *Dactylogyrus* lineages with a special focus on the origin(s) of *Dactylogyrus* parasitizing Northwest African and Iberian cyprinid fish species, and (ii) infer potential scenarios of the *Dactylogyrus* colonization of Northwest African and Iberian cyprinids in relation to their historical biogeography.

## Methods

### *Dactylogyrus* species

For this study, *Dactylogyrus* species were sampled from cyprinid species in Morocco and the Iberian Peninsula. Other *Dactylogyrus* spp. collected from cyprinid species sampled in Europe (the Balkan Peninsula, including Greece and Bosnia and Herzegovina, and central Europe, represented by the Czech Republic) and Africa (Senegal) were included in this study. These *Dactylogyrus* spp. were selected to recover representatives parasitizing different cyprinid lineages and also to include species potentially phylogenetically related to the *Dactylogyrus* spp. collected in Northwest Africa and the Iberian Peninsula. In addition, *Dactylogyrus* species from Asian cyprinid species, for which molecular data (i.e. the sequences of 28S DNA) were available in GenBank, were included in the analyses. The list of studied *Dactylogyrus* species, their host species, locality of collection, and accession numbers are presented in Table 1.

**Table 1:** List of *Dactylogyrus* species, their cyprinid host species, cyprinid phylogeny, country of collection and GenBank accession numbers for sequences used in the phylogenetic analyses

*Dactylogyrus* species	Cyprinid host species	Cyprinid subfamily	Cyprinid tribe	Country of collection	GenBank ID (28S rDNA)	GenBank ID (18S rDNA with ITS1)
*D. bicornis* Malewitzkaja, 1941^a^	*Rhodeus meridionalis* Karaman, 1924	Acheilognathinae	–	Greece	KY629345	–
*D. labei* Musselius & Gussev, 1976	*Catla catla* (Hamilton, 1822)	Cyprininae	Labeonini	India	JX566720	–
*D. quanfami* Ha Ky, 1971	*Cirrhinus molitorella* (Valenciennes, 1844)	Cyprininae	Labeonini	China	EF100536	–
*D. lenkoranoides* El Gharbi, Renaud & Lambert, 1992	*Luciobarbus guiraonis* (Steindachner, 1866)	Cyprininae	Barbini	Spain	KY629346	–
*D. bocageii* Alvarez Pellitero, Simón Vicente & González Lanza, 1981	*Luciobarbus bocagei* (Steindachner, 1864)	Cyprininae	Barbini	Portugal	KY629347	–
*D. balistae* Simón Vicente, 1981	*Luciobarbus bocagei*	Cyprininae	Barbini	Portugal	–	KY629344
*D. mascomai* El Gharbi, Renaud & Lambert, 1992	*Luciobarbus guiraonis*	Cyprininae	Barbini	Spain	KY629348	–
*D. linstowoides* El Gharbi, Renaud & Lambert, 1992	*Luciobarbus guiraonis*	Cyprininae	Barbini	Spain	KY629349	KY629329
*D. legionensis* González Lanza & Alvarez Pellitero, 1982	*Luciobarbus guiraonis*	Cyprininae	Barbini	Spain	KY629350	KY629330
*D. andalousiensis* El Gharbi, Renaud & Lambert, 1992	*Luciobarbus sclateri* Günther, 1868	Cyprininae	Barbini	Portugal	KY629351	KY629331
*D. zatensis* El Gharbi, Birgi & Lambert, 1994	*Carasobarbus fritschii* Günther, 1874	Cyprininae	Torini	Morocco	KY629352	KY629335
*D. volutus* El Gharbi, Birgi & Lambert, 1994	*Carasobarbus fritschii*	Cyprininae	Torini	Morocco	KY629353	KY629334
*D. kulindrii* El Gharbi, Birgi & Lambert, 1994	*Carasobarbus fritschii*	Cyprininae	Torini	Morocco	KY629354	KY629336
*D. marocanus* El Gharbi, Birgi & Lambert, 1994^b^	*Carasobarbus fritschii,*	Cyprininae	Torini	Morocco	KY629355	KY629333
*D. scorpius* Rahmouni, Řehulková & Šimková, 2017	*Luciobarbus rifensis* Doadrio, Casal-Lopéz & Yahyaoui, 2015	Cyprininae	Barbini	Morocco	KY553860	KY578023
*D. benhoussai* Rahmouni, Řehulková & Šimková, 2017	*Luciobarbus moulouyensis* (Pellegrin, 1924)	Cyprininae	Barbini	Morocco	KY553862	KY578025
*D. varius* Rahmouni, Řehulková & Šimková, 2017	*Luciobarbus maghrebensis* Doadrio, Perea & Yahyaoui, 2015	Cyprininae	Barbini	Morocco	KZ553863	KY578026
*D. falsiphallus* Rahmouni, Řehulková & Šimková, 2017	*Luciobarbus maghrebensis*	Cyprininae	Barbini	Morocco	KZ553861	KY578024
*D. atlasensis* El Gharbi, Birgi & Lambert, 1994	*Luciobarbus pallaryi* (Pellegrin, 1919)	Cyprininae	Barbini	Morocco	KY629356	KY629337
*D. fimbriphallus* El Gharbi, Birgi & Lambert, 1994	*Luciobarbus massaensis* (Pellegrin, 1922)	Cyprininae	Barbini	Morocco	KY629357	KY629332
*Dactylogyrus* sp. 1	*Enteromius niokoloensis* (Daget, 1959)	Cyprininae	Smiliogastrini	Senegal	KY629358	–
*D. aspili* Birgi & Lambert, 1987	*Enteromius macrops* (Boulenger, 1911)	Cyprininae	Smiliogastrini	Senegal	KY629359	–
*D. leonis* Musilová, Řehulková & Gelnar, 2009	*Labeo coubie* Rüppell, 1832	Cyprininae	Labeonini	Senegal	KY629360	–
*D. oligospirophallus* Paperna, 1973	*Labeo coubie*	Cyprininae	Labeonini	Senegal	KY629361	–
*D. brevicirrus* Paperna, 1973	*Labeo parvus* Boulenger, 1902	Cyprininae	Labeonini	Senegal	KY629362	–
*D. senegalensis* Paperna, 1969	*Labeo senegalensis* Valenciennes, 1842	Cyprininae	Labeonini	Senegal	KY629363	–
*D. titus* Guégan, Lambert & Euzet, 1988	*Labeo senegalensis*	Cyprininae	Labeonini	Senegal	KY629364	–
*D. falcilocus* Guegan, Lambert & Euzet, 1988	*Labeo coubie*	Cyprininae	Labeonini	Senegal	KY629365	–
*D. vastator* Nybelin, 1924	*Carassius gibelio* (Bloch, 1782)	Cyprininae	Cyprinini	Czech Republic	KY629366	KY201103
*D. extensus* Mueller & Van Cleave, 1932	*Cyprinus carpio* Linnaeus, 1758	Cyprininae	Cyprinini	Czech Republic	AY553629	–
*D. inexpectatus* Isjumova in Gussev, 1955	*Carassius gibelio*	Cyprininae	Cyprinini	Czech Republic	AJ969945	–
*D. anchoratus* (Dujardin, 1845)	*Carassius gibelio*	Cyprininae	Cyprinini	Czech Republic	KY201116	KY201102
*Dactylogyrus* sp. AC2012	*Cyprinus carpio*	Cyprininae	Cyprinini	India	JQ926198	–
*D. dyki* Ergens & Lucky, 1959	*Barbus barbus* (Linnaeus, 1758)	Cyprininae	Barbini	Czech Republic	KY629367	KY629338
*D. crivellius* Dupont & Lambert, 1986	*Barbus peloponesius* Valenciennes, 1842	Cyprininae	Barbini	Greece	KY629368	KY629339
*D. carpathicus* Zachvatkin, 1951	*Barbus barbus*	Cyprininae	Barbini	Czech Republic	KY201111	KY201098
*Dactylogyrus* sp. 2	*Luciobarbus albanicus* (Steindachner, 1870)	Cyprininae	Barbini	Greece	KY201114	KY201100
*Dactylogyrus* sp. 3	*Luciobarbus graecus* (Steindachner, 1895)	Cyprininae	Barbini	Greece	KY201115	KY201101
*D. prespensis* Dupont & Lambert, 1986	*Barbus prespensis* Karaman, 1924	Cyprininae	Barbini	Greece	KY201110	KY201096
*D. petenyi* Kastak, 1957	*Barbus balcanicus* Kotlík, Tsigenopoulos, Ráb & Berrebi, 2002	Cyprininae	Barbini	Greece	-	KY201097
*D. malleus* Linstow, 1877	*Barbus barbus*	Cyprininae	Barbini	Czech Republic	KY201112	KY201099
*D. vistulae* Prost, 1957	*Squalius prespensis* (Fowler, 1977)	Leuciscinae	–	Albania	KY629369	KY629640
*D. fallax* Wagener, 1857	*Vimba vimba* (Linnaeus, 1758)	Leuciscinae	–	Czech Republic	KY629370	KY629341
*D. cornu* Linstow, 1878	*Vimba vimba*	Leuciscinae	–	Czech Republic	KY629371	KY629342
*D. borealis* Nybelin, 1937	*Phoxinus* sp.	Leuciscinae	–	Bosnia and Herzegovina	KY629372	KY629343
*D. nanus* Dogiel & Bychowsky, 1934	*Rutilus rutilus* (Linnaeus, 1758)	Leuciscinae	–	Czech Republic	AJ969942	AJ564145
*D. sphyrna* Linstow, 1878	*Rutilus rutilus*	Leuciscinae	–	Czech Republic	AJ969943	AJ564154
*D. suecicus* Nybelin, 1937	*Rutilus rutilus*	Leuciscinae	–	Czech Republic	KY629373	–
*D. crucifer* Wagener, 1857	*Rutilus rutilus*	Leuciscinae	–	Czech Republic	KY629374	AJ564120
*D. wunderi* Bychowsky, 1931	*Abramis brama* (Linnaeus, 1758)	Leuciscinae	–	Czech Republic	KY629375	AJ564164
*D. cryptomeres* Bychowsky, 1943	*Gobio gobio* (Linnaeus, 1758)	Gobioninae	–	Czech Republic	AJ969947	–
*D. lamellatus* Achmerow, 1952	*Ctenopharyngodon idella* (Valenciennes, 1844)	Xenocyprinae	–	China	AY307019	–
*D. hypophthalmichthys* Akhmerov, 1952	*Hypophthalmichthys molitrix* (Valenciennes, 1844)	Xenocyprinae	–	China	EF100532	–
*Dactylogyrus* sp. (YY)	*Hypophthalmichthys nobilis* (Richardson, 1845)	Xenocyprinae	–	China	EF100538	–
*D. parabramis* Akhmerov, 1952	*Megalobrama terminalis* (Richardson, 1846)	Xenocyprinae	–	China	EF100534	–
*D. petruschewskyi* Gussev, 1955	*Megalobrama amblycephala* Yih, 1955	Xenocyprinae	–	China	AY548927	–
*D. pekinensis* Gussev, 1955	*Megalobrama amblycephala*	Xenocyprinae	–	China	EF100535	–

^a^Morphologically identical *D. bicornis* was also found on *Rhodeus amarus* (Bloch, 1782) from the Czech Republic; the sequence data are not available

^b^Morphologically and genetically identical *D. marocanus* was also collected from *Pterocapoeta maroccana*, *Luciobarbus ksibii*, *Luciobarbus zayanensis*

In the field, *Dactylogyrus* species were removed from fish gills during fish dissection (following Ergens & Lom [27]), placed on slides, covered with a coverslip, and fixed in a mixture of glycerine and ammonium picrate (GAP). The identification was performed on the basis of the size and shape of the sclerotized parts of the attachment organ, the haptor, and the sclerotized parts of the reproductive organs, following the original descriptions [11, 12, 28–32]. Morphological examination was performed using an Olympus BX51 light microscope equipped with phase contrast and differential interference contrast. Some specimens of each *Dactylogyrus* species were bisected; one-half of the body (usually the anterior one with reproductive organs) was mounted on a slide for species identification, and the other was individually preserved in 96% ethanol for DNA extraction.

### DNA extraction, amplification and sequencing

*Dactylogyrus* species collected from cyprinids in Africa and Europe were sequenced to obtain partial sequences of 28S rDNA and partial sequences of 18S rDNA and the ITS1 region. *Dactylogyrus* specimens were individually removed from ethanol and dried by using a vacuum centrifuge. Genomic DNA extraction was performed following a standard protocol (DNeasy Blood & Tissue Kit, Qiagen, Hilden, Germany). Partial 28S rDNA was amplified using the forward primer C1 (5′-ACC CGC TGA ATT TAA GCA-3′) and the reverse primer D2 (5′-TGG TCC GTG TTT CAA GAC-3′) [33]. PCR followed the protocol included in Šimková et al. [34]. Partial 18S rDNA and the entire ITS1 region were amplified in one round using the primers S1 (5′-ATT CCG ATA ACG AAC GAG ACT-3′) and IR8 (5′-GCT AGC TGC GTT CTT CAT CGA-3′) [35] that anneal to 18S and 5.8S rDNA, respectively. Each amplification reaction for partial 18S rDNA and the ITS1 region was performed in a final volume of 15 μl, containing 1.5 U of *Taq* polymerase, 1× buffer, 1.5 mM MgCl_2_, 0.2 mM of each dNTP, 0.5 μM of each primer, and 2.5 μl of DNA (20 ng/μl). PCR was carried out using the following steps: 2 min at 94 °C, followed by 40 cycles of 1 min at 94 °C, 1 min at 53 °C and 1 min 30 s at 72 °C, and 10 min of final elongation at 72 °C. The PCR products were checked on 1.5% agarose gel, purified using ExoSAP-IT kit (Ecoli, SK) following a standard protocol and directly sequenced using the PCR primers and BigDye Terminator Cycle sequencing kit (Applied Biosystems, Foster City, CA). Sequencing was carried out using an ABI 3130 Genetic Analyser (Applied Biosystems). Sequences were analysed using Sequencher 4.7 (Gene Codes Corp., Ann Arbor, MI, USA), and new sequences were deposited in GenBank (see Table 1 for accession numbers). The sequences of other *Dactylogyrus* species parasitizing European and Asian cyprinid species were retrieved in GenBank (Table 1) and were used for phylogenetic analyses.

### Phylogenetic analyses

The first alignment included the partial 28S rDNA sequences of 55 *Dactylogyrus* species. Among them, 36 were newly sequenced for this study. The sequences of the other 19 *Dactylogyrus* species as well as the sequences of three species of the Dactylogyridae (*Euryhaliotrematoides pirulum* Plaisance & Kritsky, 2004, *Euryhaliotrematoides triangulovagina* Yamaguti, 1968 and *Aliatrema cribbi* Plaisance & Kritsky, 2004 with accession numbers AY820618, AY820619 and AY820612, respectively), used as the outgroup in the phylogenetic analyses, were retrieved from GenBank. The second alignment included the partial 18S rDNA sequences and the ITS1 region of 26 *Dactylogyrus* species belonging to *Dactylogyrus* lineage III. *D. vistulae* Prost, 1957 and *D. sphyrna* Linstow, 1978 were used as the outgroup in the phylogenetic analyses based on the 18S rDNA and ITS1 sequences.

All sequences of a given dataset were aligned using ClustalW multiple alignments [36] in Bioedit v. 7.2.5 [37]. The phylogenetic analyses were performed using unambiguous alignments. Gaps and ambiguously aligned regions were removed from alignments using GBlocks v. 0.91 [38]. The best-fit DNA evolution model was determined using the Akaike’s information criterion (AIC) in JmodelTest 2.1.10 [39, 40]. Phylogenetic trees were inferred using minimum evolution (ME) analysis using PAUP* 4b10 [41], maximum likelihood (ML) analysis using PhyML 3.0 [42], and Bayesian inference (BI) analysis using MrBayes 3.2 [43]. Supports for internal nodes were computed from a bootstrap re-sampling procedure [44] with 1000 pseudoreplicates for ME, and 500 pseudoreplicates for ML using the TBR algorithm. A search for the best ML tree was performed using the TBR branch-swapping algorithm. Bayesian inference (BI) analyses were performed using four Monte Carlo Markov chains running on 1000,000 generations for each data set, with trees being sampled every 100 generations. The “burn-in” asymptote was estimated by plotting the number of generations against the log likelihood scores for the saved trees, and all the trees (25%) before stationarity were discarded as “burn-in”. The posterior probabilities of the phylogeny and its branches were determined for all trees left in the plateau phase with the best ML scores.

The mapping of characters was performed in Mesquite 3.2 [45]. Prior to the mapping of characters, a new alignment was prepared using partial 28S rDNA sequence data from 55 *Dactylogyrus* species. Phylogenetic reconstruction using BI analysis was performed as described above. *Dactylogyrus bicornis* Malewitzkaja, 1941 was used for rooting the phylogenetic tree following the output of phylogenetic analyses using the external outgroup. The first character mapped onto the phylogenetic reconstruction represents fish lineages, i.e. different fish families as applied in Yang et al. [16] (Acheilognathinae, Xenocyprinae, Gobioninae, Leuciscinae and Cyprininae as different character states). The second character represents fish lineages including the branching within Cyprininae, the target group of our study (Cyprinini, Labeonini, Torini, Smiliogastrini, Barbini including the genus *Barbus* Cuvier & Cloquet, 1816, and Barbini including the genus *Luciobarbus* were used as the character states). The revised classification of the subfamily Cyprininae by Yang et al. [16] was adopted for this mapping. The last character represents the distribution of host species with the following character states applied: southern Asia including Southeast Asia, a large part of Eurasia, Europe with only West Asia, the Iberian Peninsula, the Balkan Peninsula, Northwest Africa and West Africa. The distribution of cyprinid species follows Froese & Pauly [46].

## Results

**Fig. 1:**
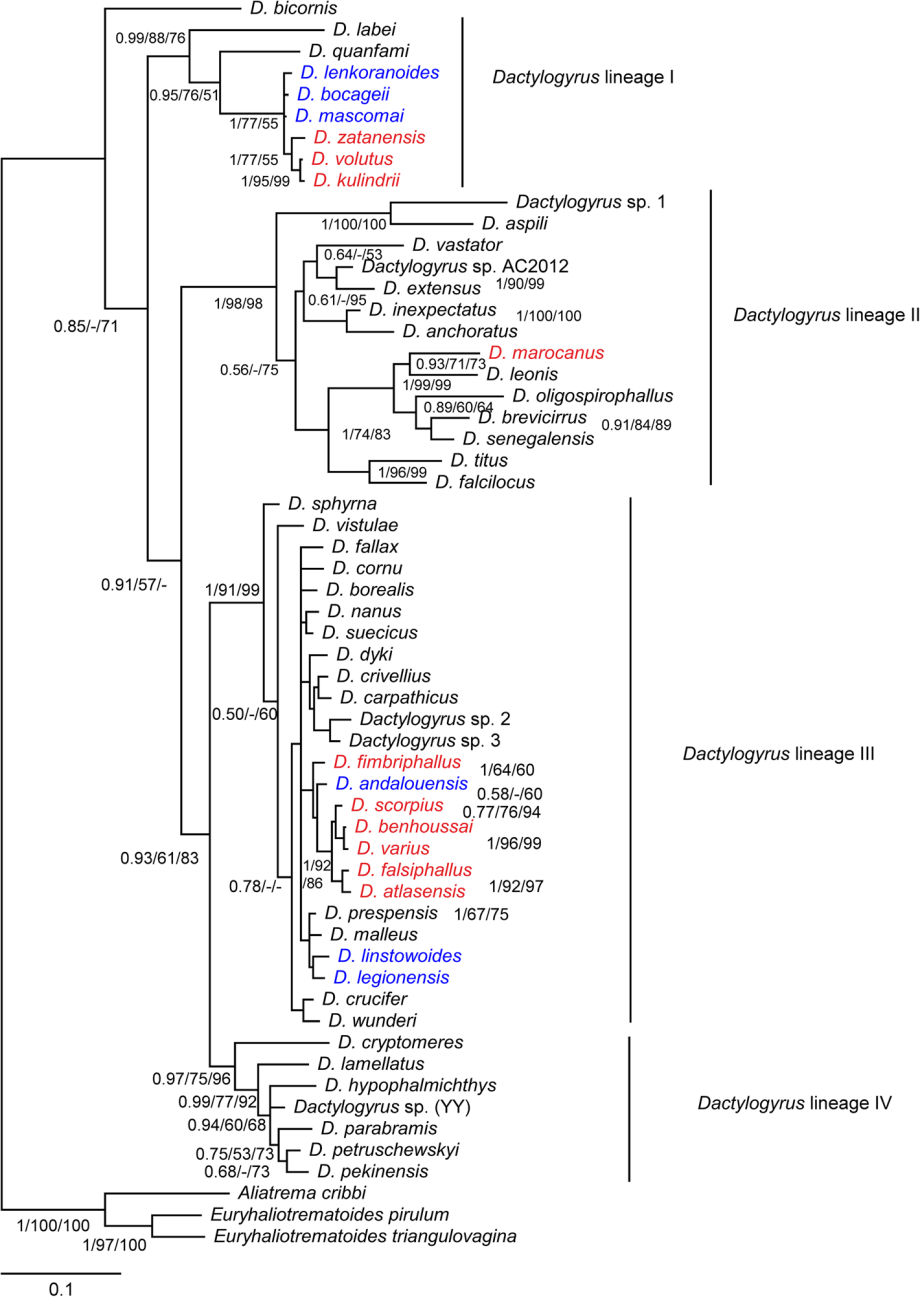
Bayesian inference phylogenetic tree of *Dactylogyrus* species parasitizing different cyprinid lineages based on sequences of partial 28S rDNA. *Dactylogyrus* spp. from Northwest African cyprinids are shown in red. *Dactylogyrus* spp. from Iberian cyprinids are shown in blue. Values along branches indicate posterior probabilities and bootstrap values resulting from the following analyses: BI/ME/ML. *Abbreviations*: BI, Bayesian inference; ME, minimum evolution; ML, maximum likelihood. *Aliatrema cribbi*, *Euryhaliotrematodes pirulum* and *E. triangulovagina* were used as the outgroup

An unambiguous alignment including the 55 *Dactylogyrus* species analysed and three outgroup species spanned 544 positions. The TVM + I + G model was selected as the best-fit evolutionary model. The ME, ML and BI analyses provided phylogenetic trees with similar topologies. The BI tree is presented in Fig. 1, including bootstrap values resulting from ME and ML analyses and posterior probabilities resulting from BI analysis. The phylogenetic reconstructions revealed four *Dactylogyrus* lineages with *D. bicornis* in the basal position (Fig. 1). *Dactylogyrus* lineage I included two *Dactylogyrus* species parasitizing Asian Labeonini in the basal position, and the monophyletic group including 3 *Dactylogyrus* parasitizing Iberian *Luciobarbus* (the tribe Barbini within Cyprininae) and *Dactylogyrus* parasitizing Northwest African *Carasobarbus fritschii* (Günther, 1874) (the tribe Torini within Cyprininae) (Table 1, Fig. 1). The other three *Dactylogyrus* lineages (II, III and IV) formed a clade well supported by BI analysis but weakly supported by ME and unsupported by ML. *Dactylogyrus* lineage II included two groups of African *Dactylogyrus*. The first group included *Dactylogyrus* parasitizing small *Enteromius* Cope, 1867 species (Smiliogastrini) collected in West Africa (the basal position of this group was weakly supported by PP resulting from BI analysis and BP resulting from ML analysis and unsupported by BP resulting from ME analysis). The second group included *Dactylogyrus* species parasitizing West African *Labeo* Cuvier, 1816 (Labeonini) with the nested position of a single *Dactylogyrus* species (*D. marocanus* El Gharbi, Birgi & Lambert, 1994) from Northwest African cyprinins of the tribes Barbini and Torini. *Dactylogyrus* lineage II also included *Dactylogyrus* species parasitizing *Cyprinus carpio* Linnaeus, 1758 and the complex of *Carassius auratus* (Linnaeus, 1758), two species of Asian origin recently widely distributed in Europe. *Dactylogyrus* lineage III included the species collected from Europe and parasitizing Leuciscinae species, *Barbus* species (Barbini, Cyprininae) with a European distribution, and the Northwest African *Luciobarus* (Table 1). Phylogenetic relationships within *Dactylogyrus* lineage III were either weakly resolved or unresolved by phylogenetic analyses. However, the monophyletic group including *Dactylogyrus* parasitizing Northwest African *Luciobarbus* species and *D. andalousiensis* El Gharbi, Renaud & Lambert, 1992 parasitizing Iberian *Luciobarbus sclateri* Günther, 1868 was either well or moderately supported by our phylogenetic analyses. *Dactylogyrus* lineage IV included *D. cryptomeres* Bychowsky, 1943 parasitizing cyprinids of Gobioninae in the basal position and the well-supported monophyletic group of *Dactylogyrus* parasitizing Asian Xenocyprinae (Table 1, Fig. 1).

**Fig. 2:**
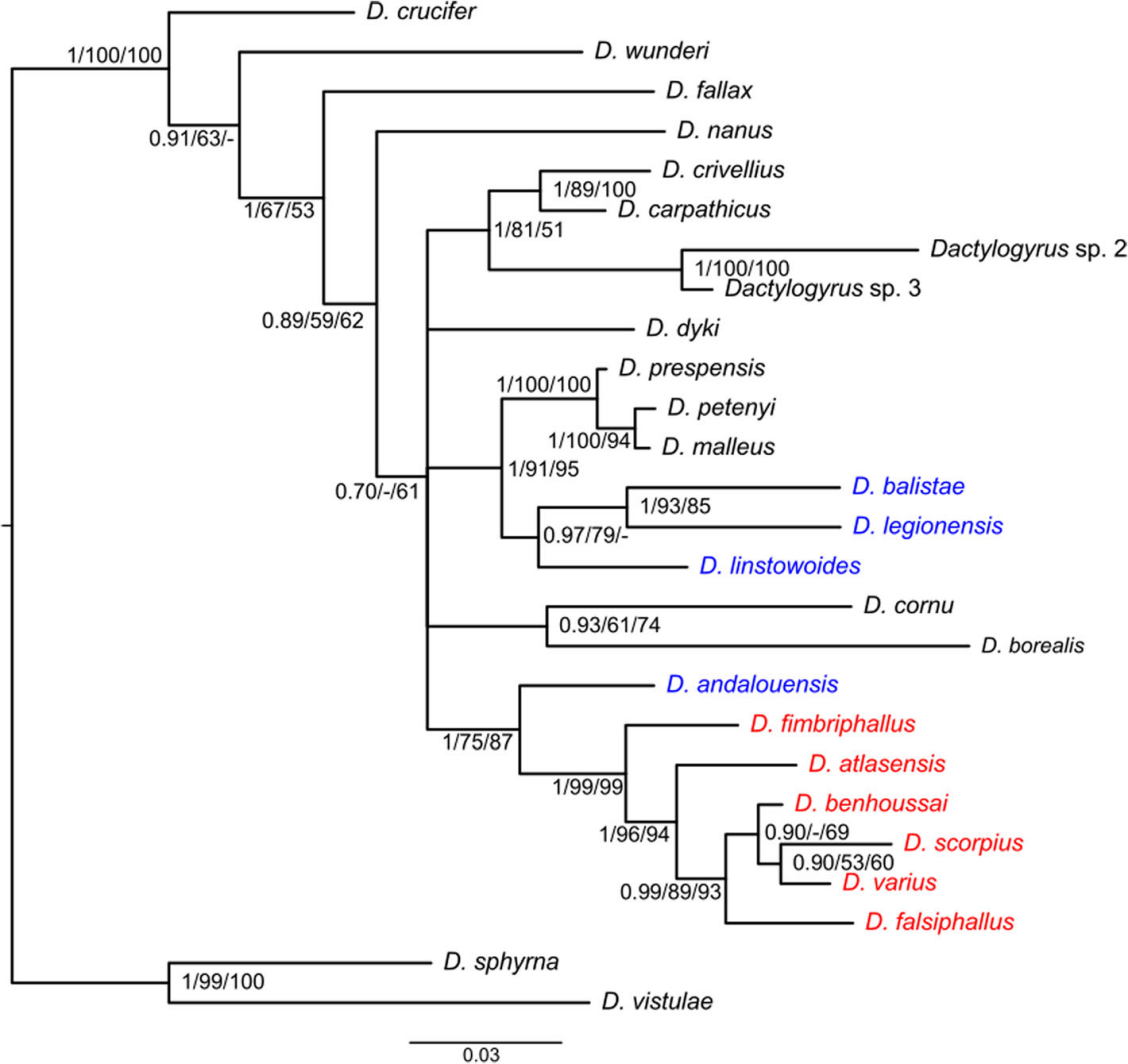
Phylogenetic tree of *Dactylogyrus* species belonging to *Dactylogyrus* lineage III constructed by Bayesian inference analysis. The tree is based on sequences of partial 18S rDNA and ITS1. *Dactylogyrus* spp. from Northwest African cyprinids are shown in red. *Dactylogyrus* spp. from Iberian cyprinids are shown in blue. Values along branches indicate posterior probabilities and bootstrap values resulting from the following analyses: BI/ME/ML. *Abbreviations*: BI, Bayesian inference; ME, minimum evolution; ML, maximum likelihood

Because of the impossibility of reconstructing a reliable alignment when including *Dactylogyrus* species of highly diversified cyprinid lineages (i.e. because of the presence of many hypervariable regions and indels), we used only the representatives of *Dactylogyrus* lineage III in subsequent phylogenetic analyses to resolve the phylogenetic relationships within this lineage. An unambiguous alignment including *Dactylogyrus* species of lineage III spanned 1072 positions. The GTR + I + G model was selected as the best-fit evolutionary model. The ME, ML and BI analyses provided phylogenetic trees with similar topologies. The BI tree is presented in Fig. 2, including bootstrap values resulting from ME and ML analyses and posterior probabilities resulting from BI analysis. The basal position of *D. andalousiensis* in relation to the monophyletic group of *Dactylogyrus* species parasitizing Moroccan *Luciobarbus* was well supported by PP resulting from BI analysis and BP resulting from ME analysis, and moderately supported by BP resulted from ML analysis. Three *Dactylogyrus* species parasitizing Iberian *Luciobarbus* species formed a monophyletic group with two *Dactylogyrus* parasitizing Balkan *Barbus* species and one *Dactylogyrus* parasitizing *Barbus* species with a wide European distribution. This cluster was well supported by all phylogenetic analyses.

**Fig. 3:**
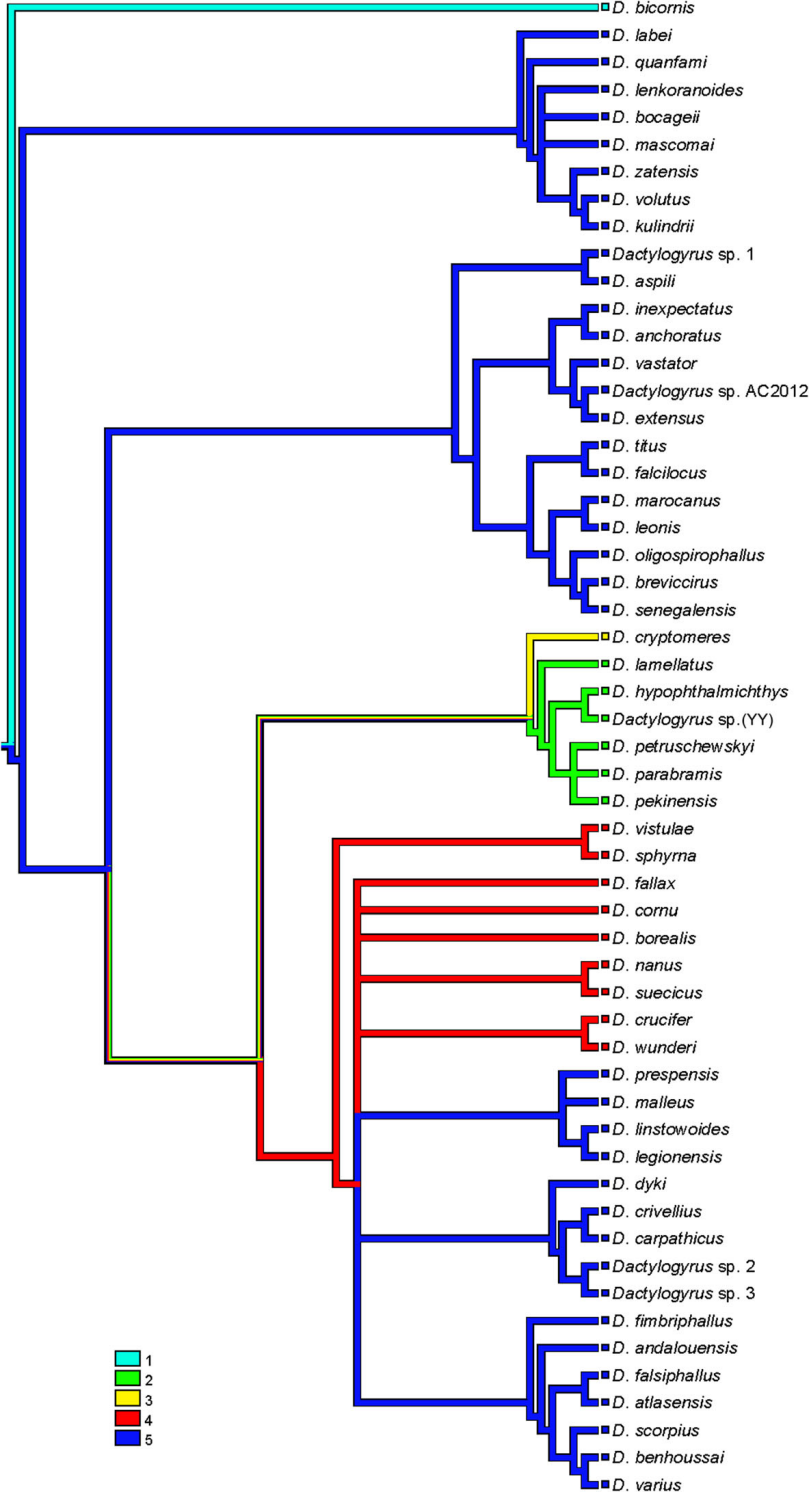
Mapping of fish lineages into the BI reconstruction of *Dactylogyrus* phylogeny. Characters for fish lineages: 1, Acheilognathinae; 2, Xenocyprinae; 3, Gobioninae; 4, Leuciscinae; 5, Cyprininae

**Fig. 4:**
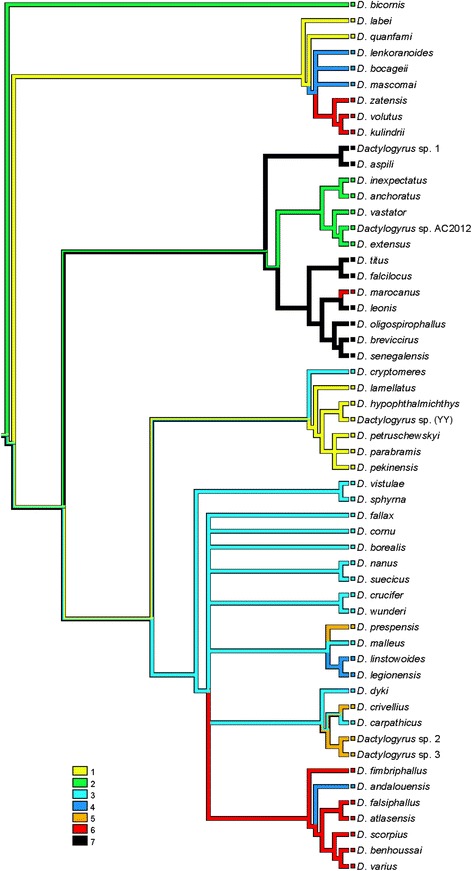
Mapping of fish distribution onto the BI reconstruction of *Dactylogyrus* phylogeny. Characters for fish distribution: 1, South and Southeast Asia; 2, Eurasia; 3, Europe with West Asia; 4, Iberian Peninsula; 5, Balkan Peninsula; 6, Northwest Africa (Mediterranean Africa); 7, West Africa. Note: The area of introduction was not considered when fish distribution was evaluated. Concerning *C. gibelio*, it is not clear whether this species is native or introduced into Europe; therefore, we retained Eurasia

**Fig. 5:**
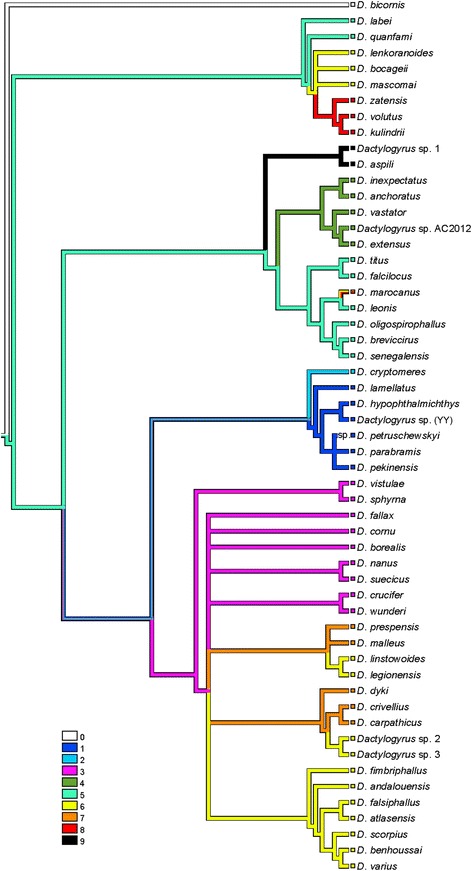
Mapping of fish lineages including detailed branching of Cyprininae into the BI reconstruction of *Dactylogyrus* phylogeny. Characters for fish lineages: 0, Acheilognathinae; 1, Xenocyprinae; 2, Gobioninae; 3, Leuciscinae; 4, Cyprininae - Cyprinini; 5, Cyprininae - Labeonini; 6, Cyprininae - Barbini - *Luciobarbus*; 7, Cyprininae - Barbini - *Barbus*; 8, Cyprininae - Torini; 9, Cyprininae - Smiliogastrini

The mapping of characters was performed in the phylogenetic reconstruction (BI tree) of 55 *Dactylogyrus* species. An unambiguous alignment spanned 568 positions. The GTR + I + G model was selected as the best evolutionary model. The mapping of the character of cyprinid lineages (i.e. cyprinid subfamilies) onto the phylogenetic reconstruction (Fig. 3) showed that Acheilognathinae is the most plesiomorphic host group for *Dactylogyrus*. *Dactylogyrus* of the Cyprininae are included in three lineages. The Gobioninae, Xenocyprinae and Leuciscinae were likely colonized by *Dactylogyrus* from the Cyprininae. However, some Cyprininae were secondarily colonized by *Dactylogyrus* from the Leuciscinae. The mapping of the cyprinid distribution onto the phylogenetic reconstruction (Fig. 4) showed the Asian origin of *Dactylogyrus*. This mapping revealed (i) the multiple origins of Northwest African *Dactylogyrus*, and (ii) the phylogenetic relatedness between *Dactylogyrus* parasitizing the Cyprininae of Labeonini, Cyprinini, Torini and some of Barbini across different continents. Northwest African *Dactylogyrus* parasitizing *Carasobarbus fritschii* (the tribe Torini within Cyprininae, see Fig. 5) are phylogenetically closely related to Asian *Dactylogyrus* species. *Dactylogyrus marocanus* is of African origin. Our mapping suggests that *D. marocanus* diverged within *Dactylogyrus* of African Labeonini and switched to Moroccan cyprinids (a morphologically and genetically identical form of this parasite was found in two species of Torini and two *Luciobarbus* species of Barbini). *Dactylogyrus* parasitizing Northwest African *Luciobarbus* are of European origin (Fig. 4). In addition, our analyses also showed the multiple origins of *Dactylogyrus* parasitizing Iberian *Luciobarbus.* The mapping of fish distribution onto *Dactylogyrus* phylogeny demonstrated that one group of *Dactylogyrus* parasitizing Iberian *Luciobarbus* (i.e. *D. mascomai* El Gharbi, Renaud & Lambert, 1992, *D. lenkoranoides* El Gharbi, Renaud & Lambert, 1992 and *D. bocageii* Alvarez Pellitero, Simón Vicente & González Lanza, 1981) and the group of *Dactylogyrus* parasitizing Northwest African Torini probably originated from Asian cyprinids (most likely Labeonini). However, the other three *Dactylogyrus* of Iberian *Luciobarbus* are most probably of European origin. Whilst *D. linstowoides* El Gharbi, Renaud & Lambert, 1992 and *D. legionensis* González Lanza & Alvarez Pellitero, 1982 form the monophyletic group with the European *Dactylogyrus* of *Barbus* species, *D. andalousiensis* is included in the monophyletic group of Northwest African *Luciobarbus* species within *Dactylogyrus* of lineage III (i.e. the lineage including *Dactylogyrus* of Leuciscinae and some *Dactylogyrus* species of *Barbus*-*Luciobarbus* group).

## Discussion

The present study was focused on host-specific monogeneans of *Dactylogyrus* as a potential tool for inferring historical contacts among their cyprinid hosts in the Mediterranean region, which is characterized by a high degree of endemism among cyprinid species. As indicated by a previous study [11, 12] and confirmed by our study, endemic Mediterranean cyprinids harbour endemic *Dactylogyrus* fauna. We investigated the origin of host-specific *Dactylogyrus* parasitizing Northwest African and Iberian cyprinid hosts, hypothesizing that phylogenetic relationships between *Dactylogyrus* species may cast new light on the biogeographical history of this fish group.

Šimková et al. [10] reconstructed the phylogeny of *Dactylogyrus* parasitizing central European cyprinid species (also including some invasive or introduced species). They presented evidence for three *Dactylogyrus* lineages in central Europe: the first includes *Dactylogyrus* of the Cyprininae (tribe Cyprinini), originating from Southeast Asia and historically introduced into Europe; the second includes *Dactylogyrus* of the Rasborinae and Xenocyprininae (the fish species of both groups originating from Southeast Asia and introduced into Europe) and the Gobioninae; and the last, a very diversified lineage, includes *Dactylogyrus* of the Leuciscinae and European *Barbus* (Barbini within Cyprininae). Šimková et al. [10] showed that the phylogenetic relationships between *Dactylogyrus* linages reflected the phylogenetic relationships between cyprinid lineages (recently represented by cyprinid subfamilies), except for the particular position of *Dactylogyrus* species parasitizing European *Barbus* species, which were nested within the highly diversified clade of *Dactylogyrus* parasitizing European Leuciscinae.

Herein, the phylogenetic position of *Dactylogyrus* parasitizing African cyprinids was evaluated for the first time. By our phylogenetic analyses, we showed that *Dactylogyrus* parasitizing the African cyprinids investigated in our study belong to three different lineages (I, II and III), which suggests their different origins and presumably also reflects the different histories of their cyprinid hosts. *Dactylogyrus* lineage II includes *Dactylogyrus* parasitizing the Cyprinini of Southeast Asian origin and West African Cyprininae (Labeonini investigated in our study), which suggests that West African cyprinids and their co-evolving *Dactylogyrus* originated from Asia (the basal position of *D. aspili* and *Dactylogyrus* sp. from small African *Enteromius* was not supported). This is in accordance with predictions on the origin of African cyprinid fauna [16, 25].

However, the situation concerning the origin of Northwest African cyprinids and their *Dactylogyrus* parasites is more complicated. *Dactylogyrus marocanus*, a single species infecting both Northwest African tribes of the Cyprininae, Torini and Barbini, was nested within *Dactylogyrus* lineage II. This parasite occurring on the representatives of two cyprinine lineages was previously reported in seven cyprinin species, mostly the representatives of Torini, by El Gharbi et al. [12] and also documented by our study. We showed a morphologically and genetically identical form of this species in *Carasobarbus fritschii*, *Pterocapoeta maroccana* Günther, 1902, *Luciobarbus ksibii* Boulanger, 1905 and *L. zayanensis* Doadrio, Casal-Lopéz & Yahyaoui, 2016. However, the abundance of *D. marocanus* was higher in two Torini species than in *Luciobarbus* species, suggesting that Torini are the main host species for its reproduction (see [9]). *Dactylogyrus marocanus* clusters within West African *Dactylogyrus* species parasitizing *Labeo* species, suggesting a single host-switch by *Dactylogyrus* to Northwest African Cyprininae from the group of Cyprininae achieving high diversification on the African continent. The attachment organ (haptor) of *D. marocanus* is of the same morphological type as that recognized for *Dactylogyrus* of West African *Labeo*, *Dactylogyrus* of small West African *Enteromius*, and two *Dactylogyrus* of Cyprinini of Southeast Asian origin i.e. *D. inexpectatus* Isjumova in Gussev, 1955 and *D. anchoratus* (Dujardin, 1845). In addition, *D. marocanus* is the only species with this type of haptor within the *Dactylogyrus* species parasitizing Northwest African cyprinids. This may suggest that haptor morphology, in this case, is a character shared by common ancestry. The similar morphology of the haptor in *Dactylogyrus* parasitizing phylogenetically closely related cyprinid species was previously demonstrated by Šimková et al. [9].

Our phylogenetic analyses using cyprinid-specific *Dactylogyrus* spp. confirmed the occurrence of different independent dispersal events from Asia (or Eurasia) to Africa concerning the Moroccan cyprinids belonging to hexaploid Torini (*Carasobarbus fritschii* and *Pterocapoeta maroccana* in our study) and tetraploid Barbini (*Luciobarbus* species), as was highlighted by the molecular phylogeny of cyprinid species [16, 17]. Middle East *Carasobarbus* and Northwest African *Carasobarbus* form a monophyletic group within the *Labeobarbus* clade, and *Pterocapoeta* occupies the basal position in this clade [16, 17]. Wang et al. [47] proposed that the group comprising the *Carasobarbus* lineage originated about 9.94 MYA in the Orient. The *Carasobarbus* lineage separated about 7.7 MYA. Tsigenopoulos et al. [17] dated the beginning of the diversification of the African hexaploid lineage to the Late Miocene following the closing of the seaway between the Mediterranean Sea and the Indian Ocean and the emergence of the Gomphotherium land bridge between Africa and Asia (the Arabian tectonic Plate) in the Middle Miocene. In the Tortonian stage, the Anatolian tectonic Plate (Asia Minor) was connected to the Arabian Plate to the east and was separated from Europe to the west, where the Aegean Sea formed [48]; this explains the absence of Torini in Europe [17]. The phylogenetic position of *Dactylogyrus* parasitizing *Carasobarbus fritschii* within *Dactylogyrus* lineage I and the phylogenetic affinity between *Dactylogyrus* species parasitizing South Asian Labeonini and *Dactylogyrus* species parasitizing Northwest African cyprinid species is in line with the hypothesis of the origin and historical dispersion of Northwest African Torini. The molecular phylogeny of tribes belonging to Cyprininae showed Labeonini to be a sister group to the group including other tribes with Torini in the basal position [16]. This may suggest close phylogenetic relationships between *Dactylogyrus* of Torini and Labeonini. However, our study suggests the need for future phylogenetic studies to investigate also the position of *Dactylogyrus* of Asian and African representatives of Torini as well as *Dactylogyrus* parasitizing other cyprinin tribes to specify the origin of *Dactylogyrus* diversity in Northwest African Torini.

Concerning the Mediterranean diversity of cyprinids, there are three main hypotheses of their historical dispersion explaining their actual distribution. All suggest that the cyprinids originated in Asia and reached the Mediterranean peninsulas via three main routes, a northern route [21], a southern route via land bridges connecting continents [25], and dispersion through the Mediterranean Sea during its supposed freshwater phase at the end of the Messinian [24]. According to the northern dispersal scenario, cyprinids dispersed slowly via river captures, through Siberia, and then from northern into southern Europe, from the late Oligocene until the late Pliocene (35–1.7 MYA). The colonization of southern Europe occurred before the alpine orogeny during the Miocene, which separated freshwater connections between northern and southern Europe [49]. Concerning *Luciobarbus*, it is hypothesized that they spread through central Europe to the Iberian Peninsula and Northwest Africa, and that, afterwards, a second invasion of *Barbus* from Asia colonized central Europe, where *Barbus* replaced *Luciobarbus* (except in the Iberian Peninsula due to the ancient isolation of the Iberian Peninsula from the rest of the European continent). This hypothesis was rejected for Iberian *Luciobarbus* by Zardoya & Doadrio [18]. According to the southern route hypothesis, cyprinids dispersed from Asia through Asia Minor via land bridges (Asian-Anatolian-Iranian, 33 MYA, and the Gomphotherium land bridge, 19 MYA) to the Balkans and Northern Africa, and subsequently to the Iberian Peninsula [24]. In accordance with this scenario, it is supposed that *Luciobarbus* colonized the Iberian Peninsula from Africa via southern Spain [18, 23]. The Lago Mare dispersal scenario [24] assumes that after the Messinian salinity crisis (5 MYA) the Mediterranean Sea underwent a lacustrine phase allowing the dispersion of freshwater fishes. This scenario predicts higher phylogenetic affinity among species in Mediterranean areas. Although this hypothesis is still widely cited, it has been largely discredited, both by geological evidence and phylogenetic studies (e.g. [19, 25]).

Recent views on the historical dispersion of *Luciobarbus* are, however, ambiguous. On the basis of morphological characters, Iberian and North African barbels are closely related to central European species, supporting the northern route of dispersion [21, 22], whilst molecular phylogenetic studies and a lack of fossil records of *Luciobarbus* in central Europe support the southern route of Iberian *Luciobarbus* dispersion [18, 19, 26]. Our phylogenetic reconstruction using host-specific *Dactylogyrus* would suggest that the northern route represents the more plausible scenario explaining the historical dispersion of *Luciobarbus* in Northwest Africa. This scenario is supported by our phylogenetic analyses, which indicate that (i) *Dactylogyrus* species parasitizing Northwest African *Luciobarbus* have a clearly European origin, and (ii) the monophyletic group of *Dactylogyrus* including *D. balistae* Simón Vicente, 1981, *D. legionensis* and *D. linstowoides* parasitizing Iberian *Luciobarbus* form a well-supported clade with *Dactylogyrus* parasitizing European *Barbus*. In addition, the Iberian species *D. andalousiensis* occupies the basal position in the clade including the monophyletic group of *Dactylogyrus* species parasitizing Northwest African *Luciobarbus* (a finding well supported by BP and PP using the combined data of partial 18S rDNA and ITS1). Even though our sampling of *Dactylogyrus* parasites did not include *Dactylogyrus* representatives of Middle East cyprinids, we showed that *Dactylogyrus* species parasitizing Northwest African *Luciobarbus*, four of the *Dactylogyrus* species parasitizing Iberian *Luciobarbus*, *Dactylogyrus* species parasitizing Greek *Luciobarbus*, *Dactylogyrus* species parasitizing Balkan *Barbus*, and *Dactylogyrus* species parasitizing the widely distributed European *Barbus barbus* form together with *Dactylogyrus* parasitizing Leuciscinae the well-supported lineage III. This may suggest the common origin of *Dactylogyrus* parasitizing *Luciobarbus*/*Barbus* (Cyprininae) of different Mediterranean areas and *Dactylogyrus* of European Leuciscinae. Indubitably, there is a strong relationship between *Dactylogyrus* parasitizing Northwest African *Luciobarbus* (Barbini) and those parasitizing European cyprinids belonging to the subfamily Leuciscinae and the tribe Barbini of the subfamily Cyprininae, identified in our *Dactylogyrus* lineage III. However, in this case, there is a large discrepancy between the phylogenies of the hosts and *Dactylogyrus* parasites, and the relationships in lineage III rather point to historical host-switching events.

In our study, we showed the close phylogenetic relationships between (i) *Dactylogyrus* parasitizing Northwest African Torini and one group of *Dactylogyrus* parasitizing Iberian *Luciobarbus* species and (ii) *Dactylogyrus* parasitizing Northwest African Barbini and the second group of *Dactylogyrus* parasitizing also Iberian *Luciobarbus* species. This revealed (i) multiple historical contacts between Iberian *Luciobarbus* and two different lineages of Northwest African cyprinids with different origins and historical dispersions, and subsequently (ii) two independent diversifications of *Dactylogyrus* in Iberian *Luciobarbus*. The exchange of fauna between the Iberian Peninsula and Northwest Africa is hypothesized for the beginning of the Messinian salinity crisis 5.96 MYA [50], which was initiated by the closing of the Betic and Rifian corridors in Spain and Morocco [51–53]. If this event was responsible for the common origin of *Dactylogyrus* parasitizing Iberian and Northwest African cyprinids, the origin and diversification of Iberian *Luciobarbus* seem to be older than predicted by the Lago Mare route of *Luciobarbus* dispersion. Mesquita et al. [54] suggested an even earlier differentiation of Mediterranean *Luciobarbus* lineages (7.3 MYA). At the end of the Messinian 5.33 MYA, all connections between North African and Iberian populations were closed by the formation of the Strait of Gibraltar [52]. However, Machordom & Doadrio [19] suggested that the Betic area was connected with the Kabilian Mountains after its isolation from the Rifian area by the Betic-Kabilian plate in the Pliocene (3.3 MYA). Cahuzac et al. [55] proposed the existence of plates also between southern Spain and the Maghreb. These plates may potentially have served as the contact zones between Iberian and North African cyprinids and may alternatively have contributed to the common ancestry of *Dactylogyrus* parasitizing Iberian *Luciobarbus* and Northwest African Torini or Barbini. However, we failed to identify any reliable resource documenting historical contacts between Iberian *Luciobarbus* and the two Moroccan cyprinid lineages.

Mesquita et al. [54] identified three polytomic evolutionary lineages of Iberian *Luciobarbus*, potentially suggesting multiple speciation events which could likely explain the evidence of two lineages for Iberian *Dactylogyrus*. However, the different positions of the two Iberian *Dactylogyrus* lineages in the phylogenetic tree have no association with the evolution and recent distribution of Iberian *Luciobarbus* (according to [54]), *L. bocagei* (Steindachner, 1864) representing the Atlantic lineage, *L. guiraonis* (Steindachner, 1866) representing the Mediterranean lineage, and *L. sclateri* representing the South-Western and South-Eastern lineage. *Dactylogyrus bocageii*, previously reported as a species endemic to Spanish *Luciobarbus* [11], was present in all three Iberian *Luciobarbus* species investigated in our study and living recently in allopatry. As indicated before, *D. andalousiensis* is a single Iberian *Dactylogyrus* species with the basal position in the clade including the monophyletic group of *Dactylogyrus* species parasitizing the Northwest African *Luciobarbus*. This parasite was previously recorded on two *Luciobarbus* species, namely *L. sclateri* and *L. microcephalus* (Almaça, 1967), both restricted to southern Portugal and Spain [11], but representing different evolutionary lineages [54].

## Conclusions

To our knowledge, this study is the first to investigate the origin and phylogenetic position of Northwest African and Iberian *Dactylogyrus*, monogenean parasites specific to cyprinid fish. The phylogenetic reconstruction of these host-specific monogeneans sheds new light on historical contacts between African and European (here Iberian) cyprinids, these contacts associated with host switches of *Dactylogyrus* parasites. More specifically, phylogenetic analyses using *Dactylogyrus* demonstrated different and independent dispersal events from Asia (or Eurasia) to Africa concerning two lineages of Moroccan cyprinids: (i) *Carasobarbus fritschii* and *Pterocapoeta maroccana* belonging to hexaploid Torini, and (ii) *Luciobarbus* species belonging to teptraploid Barbini. In addition, our study revealed that *Dactylogyrus* parasitizing Iberian *Luciobarbus* do not form a monophyletic group, i.e. we demonstrated close phylogenetic relationships between (i) *Dactylogyrus* parasitizing Northwest African Torini and one group of *Dactylogyrus* parasitizing Iberian *Luciobarbus* species, and (ii) *Dactylogyrus* parasitizing Northwest African Barbini and the second group of *Dactylogyrus* parasitizing also Iberian *Luciobarbus*. This suggests multiple historical contacts between Iberian *Luciobarbus* and Northwest African cyprinids with different origins and historical dispersions, and subsequently two independent diversification of *Dactylogyrus* in Iberian *Luciobarbus*.
